# Effects of Neighborhood Ethnic Density and Psychosocial Factors on Colorectal Cancer Screening Behavior Among Asian American Adults, Greater Philadelphia and New Jersey, United States, 2014–2019

**DOI:** 10.5888/pcd18.210062

**Published:** 2021-09-30

**Authors:** Aisha Bhimla, Tyrell Mann-Barnes, Hemi Park, Ming-Chin Yeh, Phuong Do, Ferdinand Aczon, Grace X. Ma

**Affiliations:** 1 Center for Asian Health, Lewis Katz School of Medicine, Temple University, Philadelphia, Pennsylvania; 2 Nutrition Program, Hunter College, City University of New York, New York, New York; 3 Ilocano Cultural Association of Greater Philadelphia, Cherry Hill, New Jersey; 4 Department of Clinical Sciences, Lewis Katz School of Medicine, Temple University, Philadelphia, Pennsylvania

## Abstract

**Introduction:**

We examined how neighborhood ethnic composition influences colorectal cancer (CRC) screening behavior in Asian American adults and explored whether associations between psychosocial predictors, including knowledge, self-efficacy, and barriers affecting CRC screening behavior, varied by level of neighborhood ethnic composition.

**Methods:**

Filipino, Korean, and Vietnamese Americans (N = 1,158) aged 50 years or older were included in the study. Psychosocial factors associated with CRC screening, CRC screening behavior, and sociodemographic characteristics were extracted from participants’ data. Neighborhood ethnic composition was characterized as the census-tract–level percentage of Asian residents. Participants’ addresses were geocoded to the census tract level to determine whether they resided in an ethnically dense neighborhood. Multilevel logistic regression models were run with and without interaction terms.

**Results:**

In mixed-effects logistic regression model 1, residing in an ethnically dense neighborhood was associated with lower odds of CRC screening (odds ratio [OR] = 0.65; 95% CI, 0.45–0.93; *P* = .02) after controlling for age, sex, education, ethnic group, and neighborhood socioeconomic status. Greater perceived barriers to CRC screening (OR = 0.62; 95% CI, 0.50–0.77; *P* < .001) resulted in significantly lower odds of obtaining a CRC screening, while higher self-efficacy (OR = 1.17, 95% CI, 1.11–1.23, *P* < .001) was associated with higher odds. In model 2, among those residing in a high ethnic density neighborhood, greater barriers to screening were associated with lower odds of having obtained a CRC screening (OR = 0.53; 95% CI, 0.30–0.96; *P* = .04).

**Conclusion:**

We found that residing in an ethnically dense neighborhood indicated higher disparities in obtaining CRC screenings. Future studies should examine socioeconomic and cultural disparities, as well as disparities in the built environment, that are characteristic of ethnically dense neighborhoods and assess the impact of these disparities on CRC screening behaviors.

**Summary**

**What is already known on this topic?**

Neighborhood ethnic density and composition may play a critical role in individual health behaviors, attitudes, and outcomes related to colorectal cancer (CRC).

**What is added by this report?**

Few studies have been conducted to understand whether CRC screening behavior is affected by ethnic density in Asian American neighborhoods. We examined how the neighborhood environment, specifically ethnic composition and the interplay with psychosocial factors, influences CRC screening among Asian American adults.

**What are the implications for public health practice?**

Cultural and environmental characteristics of ethnically dense neighborhoods should be considered to understand cancer risk behaviors and to develop future screening interventions.

## Introduction

Colorectal cancer (CRC) is consistently one of the most commonly diagnosed cancers among Asian American adults (1). Although the US population has experienced a decline in CRC incidence, national-level data indicate sharp rises in CRC incidence among Asian American subgroups, specifically Korean and Vietnamese American individuals, as well as among Filipina women (2,3). CRC prevalence varies within populations due to a range of influences, including but not limited to heritable, environmental, behavioral, and dietary factors (4). Literature suggests that obesity, smoking, alcohol use, and minimal physical activity are modifiable risk factors significantly associated with CRC diagnosis (1).

Obtaining regular CRC screenings and early detection reduce the risk of negative outcomes associated with CRC, including late-stage diagnosis and death (5). Existing literature has shown disparities in CRC screening rates between Asian American and non-Hispanic White people (6–8). Recent screening statistics in the National Health Interview Survey indicated that Asian American adults had the lowest fecal occult blood test, colonoscopy, and sigmoidoscopy screening rates among all racial and ethnic minority groups, at 49%, compared with 65% for non-Hispanic White and 62% for Black/African American people (5). Observed CRC screening rates are low among all Asian American ethnic groups. However, the lowest screening rates were observed among Korean Americans (7). In a systematic review, only 25% to 50% of Korean Americans had received a CRC screening, in comparison to other Asian groups and non-Hispanic White people (9). Several physical and psychosocial barriers to CRC screening are faced by Asian American adults, including low levels of English proficiency, low health literacy, and lack of access to care (6,7,10–12).

Throughout the US, urbanization, migration, and immigration have contributed to population diversity and to racial and ethnic diversity in rural, urban, and suburban communities (13). The number of Asian neighborhoods in the US increased from 412 to more than 3,000 from 1980 to 2010 (14). Asian neighborhoods consist of ethnic urban enclaves and ethnoburbs in urban and suburban areas, respectively, which have varying socioeconomic conditions (14). Among Asian subgroups, Vietnamese, Filipino, and Korean communities tend to live in ethnically dense enclaves and ethnoburbs, which can strongly influence behavioral, social, psychological, and health-seeking behaviors within and across these communities (13,15). Filipino, Vietnamese, and Korean people comprise the third, fourth, and fifth largest Asian racial groups in the US, respectively (16). New Jersey has the fourth-highest population of Asian American people of all states, and Philadelphia has the tenth-highest population of Asian American people of all US cities (17). These geographical areas have hosted immigrant enclaves, such as Little Saigon, Little Manila, Koreatown, and other Asian ethnic enclaves, with ethnic enclave areas traditionally hosting recent immigrants. Ethnoburbs serve as suburbanized areas with slightly higher socioeconomic status and stability in comparison with urban ethnic enclaves (14).

Ethnic density, defined as the proportion of racial and ethnic minority residents in a specific area, is associated with social networks and social support within communities, factors that may contribute to health-seeking behaviors (18). The ethnic density effect denotes that residents of areas with higher proportions of people from one’s own racial and ethnic group adopt healthier behaviors (18). Data on the protective effects of neighborhood ethnic density and health outcomes such as smoking, body mass index, and preterm birth (18) are mixed, with studies mainly reporting a lack of association. Few studies have assessed the effects of neighborhood ethnic density and ethnic enclaves on cancer screening behaviors among Asian American subgroups, including Vietnamese, Filipino, and Korean American. In a review by Fang and Tseng, a general inverse association was found in Asian neighborhoods between ethnic density and noninfectious cancer (eg, colorectal, breast) incidence, and a positive association was found between ethnic density and infectious cancer (eg, cervical, liver) incidence (13). Ethnic density may play a critical role in individual health behaviors, attitudes, and outcomes related to CRC and CRC screening procedures, such as colonoscopy and blood stool tests (13,19,20). Although no available literature is available specific to Asian American people and their subgroups on cancer screening behaviors, a recent study in Philadelphia found that high ethnic density and geographic segregation were associated with lower CRC screening rates in Black communities (21).

The summation of psychosocial factors such as social support, knowledge, social influence, health beliefs, and cultural norms that influence CRC screening initiation and long-term screening adherence may cause residents of ethnically dense communities with foreign-born and US-born Asian American populations to experience nuanced barriers to CRC screening (10,22). Considering the wide variability in previous research findings and lack of research that focuses exclusively on the experiences of Asian American people, we aimed to fill this gap in the literature and further examine the effects of ethnic density on CRC screening behaviors in Asian American populations in Philadelphia County, Pennsylvania, and in New Jersey. We also explored whether the associations between psychosocial predictors varied by level of ethnic density.

## Methods

### Study design and population

This cross-sectional study included participants who were part of a clustered randomized intervention to increase CRC screening in the community. We used a community-based participatory research approach aiming to explore the impact of multilevel factors on CRC screening in a sample of Filipino, Korean, and Vietnamese American adults in the Greater Philadelphia and New Jersey areas. The study had 1,158 participants aged 50 years or older from the 3 Asian American subgroups residing in Philadelphia County and New Jersey. Participants were recruited from 48 community-based organizations (CBOs) located in the Greater Philadelphia region and southern and eastern New Jersey. CBO sites consisted of religious churches and temples, adult and senior centers, and ethnic-based community centers. Data were collected from July 2014 through March 2019. 

Study participants completed a paper-based survey at baseline. The baseline survey included sociodemographic information, psychosocial predictors of CRC screening, lifestyle factors, and CRC screening history. Data on neighborhood characteristics were obtained from the 2010 US Census and the American Community Survey (ACS). Participants’ residential baseline addresses were geocoded to longitude and latitude coordinates using street centerline data to pinpoint the addresses in GIS (geographic information systems). Participants’ locations were joined with census tracts and neighborhood characteristics. A total of 86 participants’ addresses from New Jersey and 13 participants’ addresses from Philadelphia were incomplete and could not be geocoded; these were excluded from the study, leaving 1,158 participants. Participants belonged to 299 unique census tracts from the Philadelphia County and New Jersey regions.

### Measures

#### Neighborhood characteristics

**Figure 1:**
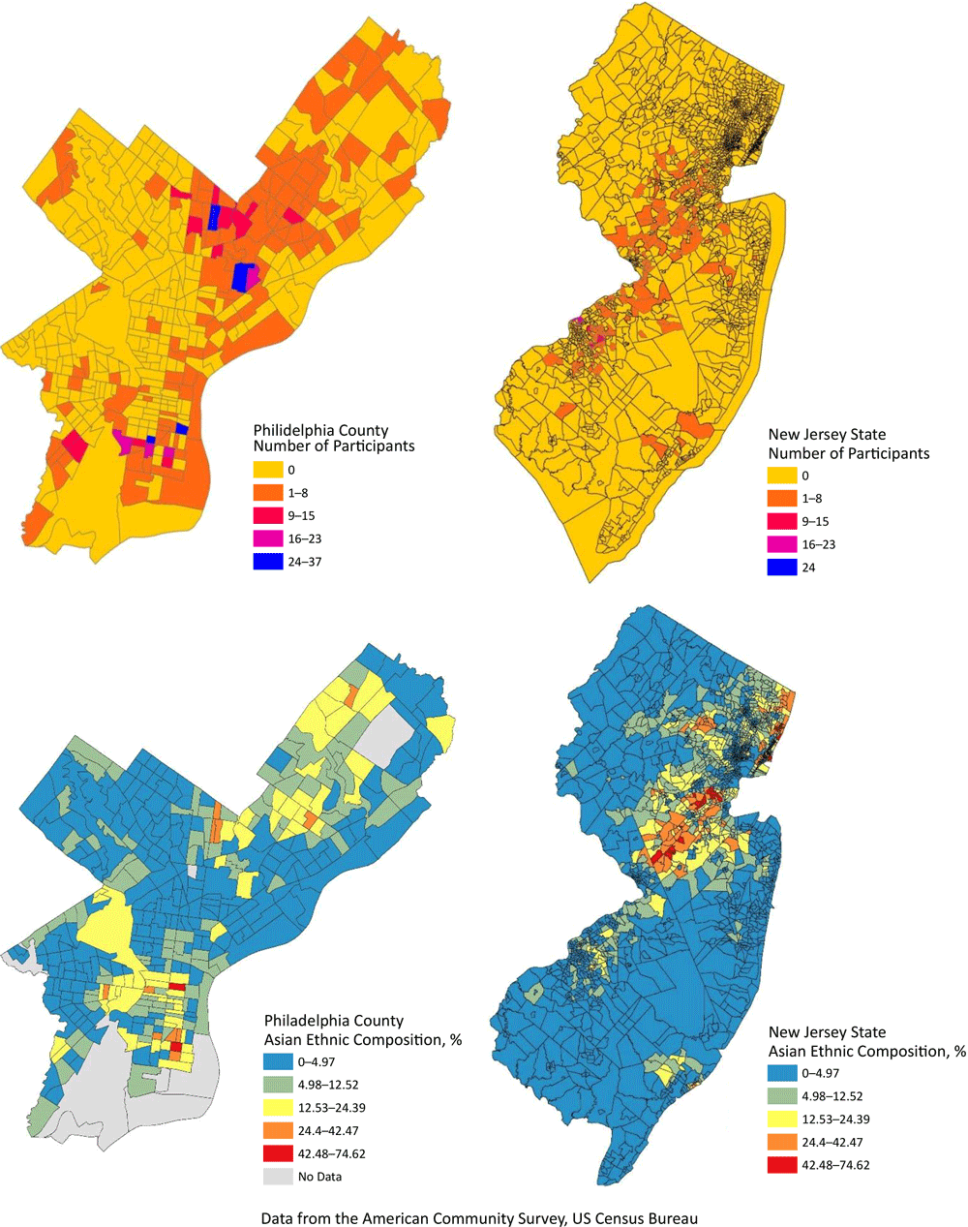
Asian ethnic composition in Philadelphia County and New Jersey census tracts. Data from the American Community Survey, US Census Bureau.

Data on ethnic density were obtained from ACS 2017 estimates and were measured by the ethnic composition of neighborhoods by obtaining the proportion of Asian American adults residing within each census tract. The density was divided into high and low, with a cut-off of the 75th percentile or above indicating high and a cut-off below the 75th percentile indicating low. Using the 75th percentile cutoff point (22.2%), 76.3% (n = 884) of the total Asian population was considered to be living in a neighborhood with low ethnic density, while the rest, 23.7% (n = 274), was considered to be living in a neighborhood with high ethnic density. Figure 1 displays the ethnic composition of neighborhoods and geographic distribution of study participants in Philadelphia County and New Jersey census tracts.

Neighborhood socioeconomic status (nSES) was assessed by obtaining 2017 ACS data on mean household income at the census tract level (23). Mean household income was included as a covariate in the model and was presented as a continuous variable.

#### Psychosocial factors

Participant’s perceived psychosocial and physical barriers to CRC screening were evaluated based on the following question: “What are the major barriers you have ever faced to obtaining a stool blood test, sigmoidoscopy, or colonoscopy?” The 3 response options were “I don’t know what it is,” “I feel healthy and do not need a sigmoidoscopy or colonoscopy,” and “I have no insurance and cannot afford it.” Each barrier was measured as 1 point, and the points were summed to obtain a barrier score (range, 0–3).

Participants were asked about their self-efficacy toward CRC screening, including whether they were confident in obtaining a screening, were able to manage emotional distress if they received a CRC diagnosis, were able to obtain information about CRC, and felt comfortable speaking to their doctor about CRC. Scores were determined using a Likert scale (0 = low self-efficacy to 10 = very high self-efficacy).

Participants’ knowledge of CRC was assessed by asking whether the following were risk factors for CRC: age, diet, family, personal history of bowel disease or CRC, sedentary lifestyle, and smoking/drinking alcohol. A response of yes was coded as 1 and a response of no was coded as 0. Scores were summed to obtain a total knowledge score (range, 0–6).

The following self-reported sociodemographic factors were collected at the individual level: sex (female, male), age, Asian origin group (Filipino, Korean, Vietnamese), education level (no education or elementary school, below high school graduate, high school graduate, some university or college), and insurance status (yes, no).

#### Outcomes

The primary outcome was individual uptake of any CRC screening modality. This measure was determined by the individual response to questions about the participant’s history of obtaining a colonoscopy or fecal occult blood test (FOBT)/fecal immunochemical test (FIT). An individual was deemed to have prior screening if he or she responded with yes to either of the screening modalities assessed with our questionnaire. A new variable was generated to reflect this convention for determining any prior CRC screening with yes being coded as 1 and no being coded as 0.

### Data analysis

Descriptive, bivariate analyses (ANOVA, analysis of variance) and logistic regression were conducted with sociodemographic, psychosocial, and neighborhood predictors. Sociodemographic variables, such as sex, Asian origin group, education, and insurance, were compared between high and low ethnic densities against CRC screening history by using bivariate analyses. These variables were further examined against the 3 psychosocial variables (barriers, knowledge, self-efficacy scores) using one-way ANOVA. Multilevel logistic regression models were used to estimate the β coefficients, odds ratios (ORs), and 95% CIs for examining neighborhood predictors of CRC screening, with a random effect for each census tract to account for the clustering of individuals (level 1) residing within neighborhoods (level 2). The multilevel logistic regression models were adjusted for sex, age, education, Asian origin group, and nSES. Model 1 presents the association between ethnic density, psychosocial predictors, and CRC screening, and model 2 presents the interaction effects between high and low ethnic density for psychosocial predictors on CRC screening history. Statistical analyses were conducted using Stata 16 (StataCorp LLC).

## Results

### Descriptive statistics of participants

The mean age of participants was 66.5 (SD, 10.0) years, and 59% (n = 678) of participants were female (Table 1). Among all participants, slightly more than half (57%, n = 655) were from the Vietnamese community, while 38% (n = 441) reported being from the Korean community and 5% (n = 62) reported being from the Filipino community. Approximately half (55%, n = 643) of participants identified zero barriers in access to medical care, while 35% (n = 400) identified 1 barrier, 8% (n = 93) identified 2 barriers, and 2% (n = 22) identified 3 barriers. The nSES measured by mean family income of the study sample was $75,143. The mean score of participant barrier knowledge was 0.56 (SD, 0.72), mean score of CRC knowledge was 1.55 (SD, 1.31), and self-efficacy was 6.38 (SD, 3.33. Overall, 31% (n = 355) reported having any CRC screening history (colonoscopy or blood stool test).

**Table 1:** Sociodemographic and Psychosocial Characteristics of Participants, by Ethnic Density, Study of the Effects of Neighborhood Ethnic Density and Psychosocial Factors on Colorectal Cancer Screening Behavior Among Asian American Adults (N = 1,158), Greater Philadelphia and New Jersey Areas, United States, 2014–2019^a^

Variable	Low Ethnic Density (≤22%)	High Ethnic Density (>23%)	Overall
**Mean barrier score (range, 0–3)^b^ (SD)**	0.56 (0.71)	0.57 (0.74)	0.56 (0.72)
**Mean knowledge score (range, 0–6)^c^ (SD)**	1.55 (1.30)	1.55 (1.33)	1.55 (1.31)
**Mean self-efficacy score (range, 0–10)^d^ (SD)**	6.45 (3.30)	6.16 (3.40)	6.38 (3.33)
**Neighborhood SES, mean income (SD), $**	74,165 (41,403)	78,297 (52,940)	75,143 (44,414)
**Age, mean (SD), y**	66.4 (10.0)	66.8 (9.7)	66.5 (10.0)
**Sex**			
Female	522 (59.3)	156 (57.1)	678 (58.8)
Male	357 (40.6)	117 (42.9)	474 (41.2)
**Asian origin group**			
Korean	325 (36.8)	116 (42.3)	441 (38.1)
Vietnamese	501 (56.7)	154 (56.2)	655 (56.5)
Filipino	58 (6.6)	4 (1.5)	62 (5.4)
**Education**			
No education or elementary school	121 (14.2)	33 (12.3)	154 (13.7)
Below high school graduate	109 (12.8)	35 (13.0)	144 (12.8)
High school graduate	323 (37.8)	102 (37.9)	425 (37.8)
University or some college	302 (35.3)	99 (36.8)	401 (35.7)
**Insurance**			
Yes	627 (76.6)	191 (74.0)	818 (76.0)
No	191 (23.4)	67 (26.0)	258 (24.0)
**CRC screening history (colonoscopy or FOBT/FIT)**			
Yes	284 (34.1)	71 (27.2)	355 (32.4)
No	549 (65.9)	190 (72.8)	739 (67.6)

Abbreviations: CRC, colorectal cancer; FOBT/FIT, fecal occult blood test/fecal immunochemical test; SES, socioeconomic status.

a Values are no. (%) unless otherwise indicated.

b Barriers to CRC screening were assessed with the following question: “What are the major barriers you have ever faced to obtaining a stool blood test, sigmoidoscopy, or colonoscopy?” The 3 response options were “I don’t know what it is,” “I feel healthy and do not need a sigmoidoscopy or colonoscopy,” and “I have no insurance and cannot afford it.” Each barrier was measured as 1 point, and scores were summed to obtain a total barriers score (range, 0–3).

c Participants were asked whether the following were risk factors for CRC: age, diet, family, personal history of bowel disease or CRC, sedentary lifestyle, and smoking/drinking alcohol. A response of yes was coded as 1 and a response of no was coded as 0. Scores were summed to obtain a total knowledge score (range, 0–6).

d Participants’ self-efficacy was assessed with the following measures: whether they were confident in obtaining a screening, whether they were able to manage emotional distress if they received a CRC diagnosis, whether they were able to obtain information about CRC, and whether they felt comfortable speaking to their doctor about CRC. Scores were determined using a Likert scale (0 = low self-efficacy to 10 = very high self-efficacy).

### Bivariate assessment of potential confounders

Asian origin group and education were identified as potential confounders through binomial regression. We found significant differences among the 3 communities (Vietnamese, Korean, Filipino) in knowledge scores (*F*_1,1156_ = 89.61, *P* < .001) and in self-efficacy scores (*F*_1,1156_ = 163.1, *P* < .001). We found significant differences among the 3 education levels (below high school graduate, high school graduate, university or some college) in barrier scores (*F*_3,1120_ = 9.618, *P* < .001), and in self-efficacy scores (*F*_3,1120_ = 4.005, *P* = .008).

### Regression analyses with predictors and moderators associated with CRC

After adjusting for Asian origin group, education, age, sex, and nSES, results showed that Asian American adults (n = 1,158 after adjusting for missing data) who lived in a neighborhood with high Asian ethnic density had significantly lower odds of having completed CRC screening (OR = 0.65; 95% CI, 0.45–0.93; *P* = .02) (Table 2). A significant association was also observed between CRC screening behavior and participant barrier scores. For each 1-unit increase in barrier score, the odds of CRC screening completion were reduced by 38% (OR = 0.62; 95% CI, 0.50–0.77, *P* < .001). In other words, the higher the barrier score, the less likely that participants had completed screening. Although not significant, a 1-unit increase in knowledge score was associated with 1.09 times greater odds of CRC screening completion (95% CI, 0.97–1.23; *P* = .14). For every 1-unit increase in self-efficacy score, the odds of CRC screening completion increased 1.17 times (95% CI, 1.11–1.23; *P* < .001). A 1-unit increase in age (OR = 1.02; 95% CI, 1.01–1.04; *P* = .005) was associated with a greater likelihood of CRC screening, while not graduating from high school (OR = 0.44; 95% CI, 0.24–0.81; *P* = .009), being Vietnamese (OR = 0.18; 95% CI, 0.12–0.27; *P* < .001), and being Filipino (OR = 0.40; 95% CI, 0.21–0.75; *P* = .005) were associated with a lower odds of CRC screening.

**Table 2:** Mixed-Effects Logistic Regression, With Psychosocial Predictors and Interaction Terms, in Predicting CRC Screening Among Asian American Adults (N = 1,158), Greater Philadelphia and New Jersey Areas, United States, 2014–2019

Variable	Model 1^a^		Model 2^b^	
	OR (95% CI)	*P* Value	OR (95% CI)	*P* Value
**High ethnic density (reference group, low)**	0.65 (0.45–0.93)	.02	0.33 (0.08–1.29)	.11
**Neighborhood mean income**	1.00 (0.99–1.00)	.98	0.99 (0.99–1.00)	.65
**Barrier score**	0.62 (0.50–0.77)	<.001	0.70 (0.55–0.90)	.004
**Knowledge score**	1.09 (0.97–1.23)	.14	1.07 (0.94–1.21)	.34
**Self-efficacy score**	1.17 (1.11–1.23)	<.001	1.16 (1.10–1.23)	<.001
**Age**	1.02 (1.01–1.04)	.005	1.02 (1.01–1.04)	.004
**Male sex (reference group, female)**	1.06 (0.70–1.27)	.71	1.05 (0.77–1.41)	.77
**Education (reference group, below elementary)**				
Below high school graduate	0.44 (0.24–0.81)	.009	0.44 (0.24–0.82)	.009
High school graduate	0.75 (0.48–1.19)	.22	0.75 (0.48–1.20)	.23
University or some college	0.86 (0.52–1.44)	.58	0.86 (0.51–1.46)	.58
**Asian origin group (reference group, Korean)**				
Vietnamese	0.18 (0.12–0.27)	<.001	0.18 (0.12–0.27)	<.001
Filipino	0.40 (0.21–0.75)	.005	0.41 (0.22–0.78)	.006
Ethnic density*barrier score	—	—	0.53 (0.30–0.96)	.04
Ethnic density*knowledge score	—	—	1.15 (0.86–1.54)	.35
Ethnic density*self-efficacy score	—	—	1.06 (0.93–1.19)	.40

Abbreviations: —, not assessed; nSES, neighborhood socioeconomic status.

a Model 1 variables: ethnic density, nSES, barriers, knowledge, self-efficacy, age, sex, education, Asian origin group.

b Model 2 variables: ethnic density, nSES, barriers, knowledge, self-efficacy, age, sex, education, Asian origin group, ethnic density*barrier, ethnic density*knowledge, ethnic density*self-efficacy.

Multiple logistic regression analyses after adjustment for Asian origin group, education, age, sex, and nSES were performed to assess interaction effects between high and low ethnic density for psychosocial predictors on CRC screening history. Ethnic density did not moderate the relationship between knowledge (OR = 1.15; 95% CI, 0.86–1.54; *P* = .35) or self-efficacy (OR = 1.06; 95% CI, 0.93–1.19; *P* = .40) and colorectal cancer screening behavior (Table 2).

**Figure 2:**
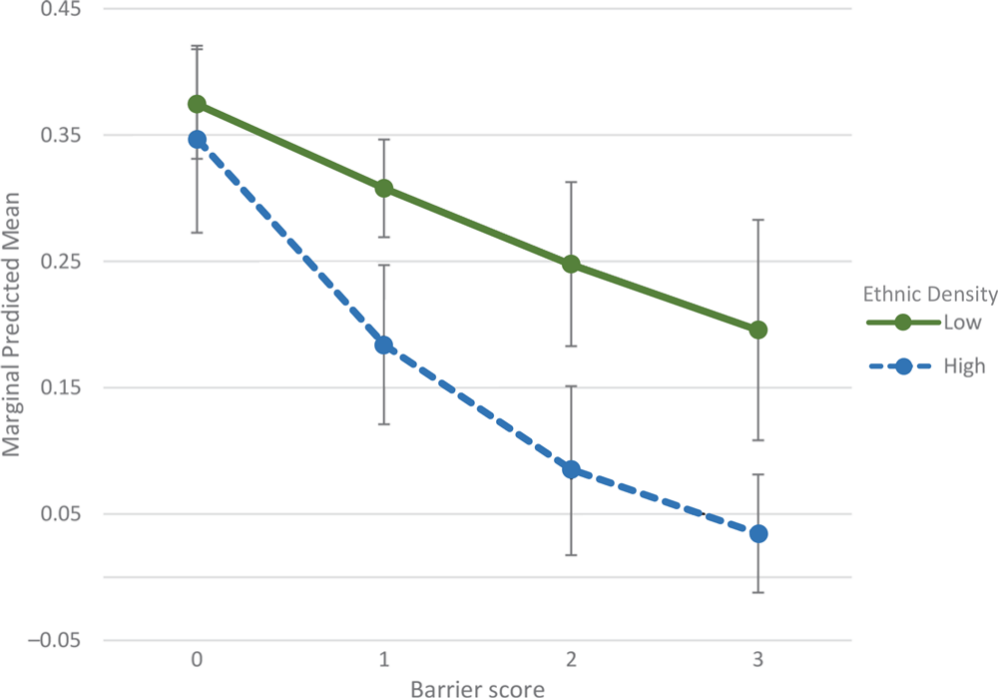
Interaction effects of high ethnic density and low ethnic density groups for barrier score on colorectal cancer (CRC) screening behavior. Three perceived barriers to CRC screening were assessed, each totaling 1 point, and summed to produce the barrier score (range, 1–3). Error bars represent 95% CIs.

The effect of neighborhood ethnic density on CRC screening history was significantly dependent on an individual’s barrier score (OR = 0.53; 95% CI, 0.30–0.96; *P* = .04). CRC screening completion rates were similar when no barriers were identified (Figure 2). However, the more barriers that an individual identified, the more that living in a high ethnic density neighborhood negatively affected CRC screening completion.

**Table:** 

Ethnic Density	Barrier Score, 95% CI			
	0	1	2	3
Low	0.37 (0.33–0.42)	0.31 (0.27–0.35)	0.25 (0.18–0.31)	0.19 (0.11–0.28)
High	0.35 (0.27–0.42)	0.18 (0.12–0.25)	0.08 (0.02–0.15)	0.03 (–0.01 to 0.08)

## Discussion

We aimed to determine the relationship between ethnic density and CRC screening among a sample of Asian individuals residing in Philadelphia and New Jersey. We found that residing in an ethnically dense Asian neighborhood was associated with negative CRC screening history without interaction terms introduced into the model. Asian American adults living in ethnically dense neighborhoods had 35% lower odds of being screened compared with those living in lower ethnic density neighborhoods when controlling for nSES. When barrier score was added as an interaction term in model 2, we found a significant effect of ethnic density on CRC screening, depending on an individual’s barrier score. When participants reported having no barriers at all, the odds for CRC screening completion in both low and high ethnic density groups were similar (0.35). However, the more barriers an individual identified, the more that living in an ethnically dense neighborhood negatively affected screening completion. These observations indicate that a dose–response effect may be present, with this psychosocial factor playing a moderating role. For instance, in high ethnic density neighborhoods, an individual who reported the maximum number of barriers had screening odds of 0.05. On the other hand, in low ethnic density neighborhoods, an individual reporting the same barriers had much higher odds of CRC screening at 0.25.

Cultural factors, such as cultural norms and beliefs, may be pertinent to ethnically dense neighborhoods and may comprise the mechanism at play (13). For example, individual barriers related to screening consisted of a lack of insurance, knowledge, and perceived health regarding CRC, all of which may be affected by cultural factors. More specifically, cultural beliefs surrounding screening, such as traditional beliefs regarding fatalism, have been reported to have adverse effects on health behaviors and have been linked to a lower adherence to screening in ethnic minority communities (8,13,24). Stigma, fatalism, and negative cultural attitudes toward cancer reinforce pre-existing barriers to screening. Further, literature suggests that there are intergenerational differences in observed cancer screening behaviors, influenced by the length of residence in the US and level of acculturation or adjustment (25). Ultimately, these systemic and social factors affect CRC screening intention and behavior, offering a sociopolitical explanation to our study results. Furthermore, individuals from ethnically dense communities are more likely to be underinsured or lack access to insurance (13). The interplay of structural barriers to screening, cultural norms, and residing in ethnically dense neighborhoods calls for efforts to better identify cultural factors to promote CRC screening. A need also exists to distinguish between ethnoburbs and ethnic enclaves to provide a greater understanding of potential moderating effects on CRC screening.

Ethnic density and ethnic neighborhood composition are associated with long-term health outcomes, access to care, and cancer screening behaviors (1). Cultural and socioeconomic factors are inextricably linked to long-term access to care, social determinants of health, and health outcomes among ethnic minorities. Although no previous research has specifically investigated the effect of ethnic density on CRC screening, studies have found that living in ethnically dense Asian or immigrant neighborhoods is associated with greater odds of late-stage CRC diagnoses and that this in turn is closely related to reduced CRC screening (26,27). Albeit a different racial group, a recent study conducted in Philadelphia found that high racial density was associated with lower rates of CRC screening in Black participants (21).

Our data suggest that Asian American adults living in ethnically dense communities are statistically less likely to have completed CRC screening. Among the Korean, Vietnamese, and Filipino American participants in our study, the CRC screening completion rate was 32%. In 2016, self-reported rates of CRC screening in the general population were 62% in Philadelphia and 65% in New Jersey (28,29). In our study, Korean American adults had the highest proportional rate of CRC screening, at 49%, compared with 47% in Filipino and 22% in Vietnamese communities. In the regression models, we found differences in the screening odds between Vietnamese, Filipino, and Korean American subgroups; compared with Korean American adults, Vietnamese American adults and Filipino American adults were 82% and 60% less likely to have completed CRC screenings, respectively. Our findings are contrary to those of other studies, such as those conducted by Hwang (30) and Juon et al (31), which found that Korean Americans had the lowest rates for screening among Asian American subgroups, specifically in the Baltimore–Washington Metropolitan area. Although the authors attributed the level of education and knowledge to such differences, the implications require confirmation through epidemiologic studies that are specifically designed to study differences between Asian American subgroups.

### Strengths and limitations

This study was among the first to examine interactions between psychosocial factors and ethnic density as predictors of CRC screening in Asian American subgroups. We used primary data collected from neighborhoods representing immigrant communities. Given that this survey was administered in multiple languages, we were able to capture non–English-speaking participants. This study has several limitations. We collected data from a convenience sample of Asian American adults residing in Philadelphia County and the state of New Jersey, so the findings may not be generalizable to a population-based, randomized stratified sample of all Asian American populations within the observed geographic area. Therefore, our findings should be interpreted with consideration of local social and cultural contexts. In addition, our study did not include several Asian American subgroups, such as Chinese, Cambodian, Indian, and Indonesian Americans, that also account for the total Asian American population in the area of interest; also, we did not include nativity as a variable in the analysis. Moreover, ethnic density measured by using census tracts may not exactly correspond to individuals’ perceived boundaries and perceptions of their neighborhood, and CRC screening completion does not necessarily indicate one’s adherence to national CRC screening guidelines. Lastly, our study relied on self-reported data, including CRC screening history, which can be subject to recall and social desirability bias.

### Future research recommendations

Disaggregating data to identify specific barriers, needs, and norms through an intercommunal and an intrapersonal lens is a critical need. Immigration data suggest that although Chinese American people make up a large portion of Asian people in the US, there is still in-group heterogeneity that influences education, socioeconomic status, and occupation (32). In-group heterogeneity is also observed among Asian sub-ethnic groups, including but not limited to Korean, Filipino, and Vietnamese American people (32). In accordance with class assimilation theory, individual social opportunity and development drives divergence from temporary reliance on the ethnic enclave or community for support (32). 

In our analysis, we observed a relationship between high ethnic density in Asian American populations and negative CRC screening behaviors. It is imperative that future studies and interventions further assess intracommunity beliefs to identify differences in generational cohorts, socioeconomic status, and degree of assimilation within each subethnic group. Future studies could assess ethnic enclaves using mixed-methods research to identify these characteristics. Our study findings support the development of personalized and culturally informed CRC interventions that focus on ethnically dense neighborhoods as a study population.
